# Differential Striatal Axonal Arborizations of the Intratelencephalic and Pyramidal-Tract Neurons: Analysis of the Data in the MouseLight Database

**DOI:** 10.3389/fncir.2019.00071

**Published:** 2019-11-15

**Authors:** Kenji Morita, Sanghun Im, Yasuo Kawaguchi

**Affiliations:** ^1^Physical and Health Education, Graduate School of Education, The University of Tokyo, Tokyo, Japan; ^2^International Research Center for Neurointelligence (WPI-IRCN), The University of Tokyo, Tokyo, Japan; ^3^Division of Cerebral Circuitry, National Institute for Physiological Sciences, Okazaki, Japan; ^4^Department of Physiological Sciences, SOKENDAI (Graduate University for Advanced Studies), Okazaki, Japan

**Keywords:** neocortex, striatum, corticostriatal, intratelencephalic, pyramidal-tract, axon arborization, morphology, MouseLight

## Abstract

There exist two major types of striatum-targeting neocortical neurons, specifically, intratelencephalic (IT) neurons and pyramidal-tract (PT) neurons. Regarding their striatal projections, it was once suggested that IT axons are extended whereas PT axons are primarily focal. However, subsequent study with an increased number of well-stained extended axons concluded that such an apparent distinction was spurious due to limited sample size. Recent work using genetically labeled neurons reintroduced the differential spatial extent of the striatal projections of IT and PT neurons through population-level analyses, complemented by observations of single axons. However, quantitative IT vs. PT comparison of a large number of axons remained to be conducted. We analyzed the data of axonal end-points of 161 IT neurons and 33 PT neurons in the MouseLight database (http://ml-neuronbrowser.janelia.org/). The number of axonal end-points in the ipsilateral striatum exhibits roughly monotonically decreasing distributions in both neuron types. Excluding neurons with no ipsilateral end-point, the distributions of the logarithm of the number of ipsilateral end-points are considerably overlapped between IT and PT neurons, although the proportion of neurons having more than 50 ipsilateral end-points is somewhat larger in IT neurons than in PT neurons. Looking at more details, among IT subpopulations in the secondary motor area (MOs), layer 5 neurons and bilateral striatum-targeting layer 2/3 neurons, but not contralateral striatum-non-targeting layer 2/3 neurons, have a larger number of ipsilateral end-points than MOs PT neurons. We also found that IT ipsilateral striatal axonal end-points are on average more widely distributed than PT end-points, especially in the medial-lateral direction. These results indicate that IT and PT striatal axons differ in the frequencies and spatial extent of end-points while there are wide varieties within each neuron type.

## Introduction

There exist two major types of striatum targeting neurons in the neocortex, specifically, intratelencephalic (IT) neurons, which project only to telencephalic regions, and pyramidal-tract (PT) neurons, which project out of telencephalon (Wilson, 1986, 1987; Cowan and Wilson, 1994; Levesque et al., 1996; Parent and Parent, 2006; Reiner et al., 2010; Shepherd, 2013). These neuron types commonly exist in neocortical areas including the motor cortices, and have distinct, albeit overlapped, layer distributions. They also have differential dendritic morphology and intracortical connectivity (Morishima and Kawaguchi, 2006; Brown and Hestrin, 2009; Morishima et al., 2011; Kiritani et al., 2012), as well as gene expression (Arlotta et al., 2005; Molyneaux et al., 2009; Tasic et al., 2018). Regarding the striatal projections of IT and PT neurons, contralateral projections arise only from IT neurons. Moreover, it was once suggested that IT axonal arborizations are extended whereas PT axons are primarily focal, based on intracellular labeling of IT and PT neurons identified by antidromic activation from the contralateral striatum (contra-Str) or the medullary pyramid, respectively (Cowan and Wilson, 1994). However, subsequent study from the same laboratory examined an increased number of well-stained extended axons (10 IT neurons and 6 PT neurons), and concluded that such an apparent difference in the axonal morphology was spurious due to limited sample size (Zheng and Wilson, 2002).

Recent work (Hooks et al., 2018) systematically examined the striatal projections of IT and PT neurons by injecting Cre-dependent fluorescent reporters into various cortical sites in mouse lines specifically expressing Cre in either IT or PT neurons (Gerfen et al., 2013). Along with revealing the neuron type- and cortical area-dependent topographic precision, which was the main focus of the study, the authors have shown that cortical injection of Cre-dependent reporter in the PT-Cre mouse line caused fewer striatal voxels with supra-threshold fluorescence intensity (Hooks et al., 2018), indicating that PT projections are spatially more limited at the population level. Moreover, they complemented their argument by observing axonal arborizations of IT and PT neurons collected through the MouseLight project at Janelia Research Campus, which performed whole brain reconstructions (Economo et al., 2016). Based on the observation, they mentioned that IT axons were more extensive and PT axons were more focal, and they also referred to the previous study (Cowan and Wilson, 1994) [but not (Zheng and Wilson, 2002)]. However, quantitative IT vs. PT comparison of a large number of axonal morphology data was not performed.

The MouseLight project has now developed the MouseLight database (http://ml-neuronbrowser.janelia.org/), which contains reconstructed morphology data of about 1000 neurons and is open to public (Economo et al., 2019; Winnubst et al., 2019). The article introducing this database (Winnubst et al., 2019) performed several analyses including those for IT and PT neurons, which revealed great diversity of IT neuronal projection patterns and also PT neuronal subtypes projecting to distinct targets. However, quantitative comparison of intra-striatal axons between IT and PT neurons was not reported. Because this long-standing issue is critical in elucidating the functions of corticostriatal (CS) circuits, we addressed it by using the MouseLight database. A limitation we faced with was that information of synapses, i.e., presynaptic terminals, was actually not available in this database. Therefore we needed to examine other available data that could be a proxy for the information about synapses, such as the length of axons in the striatum or the number and locations of axonal end-points, the latter of which we focus on in the present article. If axons entering the striatum traverse several distances before arriving at targeting sites where many synapses are made, the traversing parts of axons should contribute to the length of axons but should not affect the statistics of axonal end-points. Previous work (Anderson et al., 2002) has shown that, in cat visual cortex, the density of boutons in the most distal and second most distal axonal segments (i.e., those nearest and second nearest to the end-points) is twice higher than the density in the more proximal segments for the axons of spiny or thalamic neurons. If similar rules apply to the CS axons, our focus on the end-points could be supported.

## Methods

### Identification of PT and IT Neurons in the MouseLight Database

The source of the data used in this article is the MouseLight project at Janelia, and the DOIs of data entities are listed in Table 1. We searched entities of PT type CS neurons in the Neuron Browser of the MouseLight database (http://ml-neuronbrowser.janelia.org/) by setting three filters: (1) soma is located in the cerebral cortex (“Cerebral cortex” in the search box), (2) axonal end-point exists in the striatum (specifically, “Striatum,” “Striatum dorsal region,” “Striatum ventral region,” “Caudoputamen” or “Nucleus accumbens”; threshold: “any”), and (3) axon exists in the pons (specifically, “Pons,” “Pons, sensory related,” “Pons, motor related,” or “Pons, behavioral state related”; threshold: “any”). As a result, we found 36 entities of neocortical neurons with all the structures; 3 entities with “axon only” and a subiculum neuron were omitted from our analyses. In these 36 entities, 33 entities have soma in layer 5 whereas 2 have soma in layer 2/3 and 1 lacks description of soma layer. Since PT neurons have been described to have soma in layer 5 in previous studies, we included the 33 neurons with soma in layer 5 into analyses as PT neurons.

**TABLE 1:** List of ID number and DOI of each neuron data entity used in this article.

**ID**	**DOI**
**PT in the primary motor area (MOp)**	
AA0132	10.25378/janelia.5527270
AA0135	10.25378/janelia.5527279
AA0261	10.25378/janelia.5527717
AA0584	10.25378/janelia.7649918
AA0587	10.25378/janelia.7649927
AA0617	10.25378/janelia.7655711
AA0923	10.25378/janelia.7803737
AA0927	10.25378/janelia.7803770
**PT in the secondary motor area (MOs)**	
AA0011	10.25378/janelia.5521615
AA0012	10.25378/janelia.5521618
AA0122	10.25378/janelia.5527240
AA0180	10.25378/janelia.5527441
AA0181	10.25378/janelia.5527444
AA0182	10.25378/janelia.5527447
AA0245	10.25378/janelia.5527657
AA0250	10.25378/janelia.5527678
AA0415	10.25378/janelia.7614212
AA0583	10.25378/janelia.7649900
AA0726	10.25378/janelia.7707194
AA0764	10.25378/janelia.7710065
AA0780	10.25378/janelia.7739285
AA0788	10.25378/janelia.7739369
AA0791	10.25378/janelia.7739399
AA0792	10.25378/janelia.7739402
**PT in other areas**	
AA0001	10.25378/janelia.5520037
AA0066	10.25378/janelia.5521807
AA0119	10.25378/janelia.5526736
AA0121	10.25378/janelia.5527237
AA0796	10.25378/janelia.7739525
AA0941	10.25378/janelia.7804004
AA0944	10.25378/janelia.7804028
AA0945	10.25378/janelia.7804034
AA0956	10.25378/janelia.7804088
**IT in MOp**	
AA0002	10.25378/janelia.5520049
AA0004	10.25378/janelia.5520205
AA0010	10.25378/janelia.5521600
AA0034	10.25378/janelia.5521684
AA0035	10.25378/janelia.5521690
AA0042	10.25378/janelia.5521714
AA0062	10.25378/janelia.5521789
AA0064	10.25378/janelia.5521798
AA0065	10.25378/janelia.5521801
AA0099	10.25378/janelia.5526676
AA0102	10.25378/janelia.5526685
AA0107	10.25378/janelia.5526700
AA0108	10.25378/janelia.5526703
AA0130	10.25378/janelia.5527264
AA0140	10.25378/janelia.5527297
AA0184	10.25378/janelia.5527453
AA0271	10.25378/janelia.5527762
AA0272	10.25378/janelia.5527765
AA0289	10.25378/janelia.5527822
AA0401	10.25378/janelia.7614125
AA0404	10.25378/janelia.7614134
AA0408	10.25378/janelia.7614173
AA0440	10.25378/janelia.7614341
AA0442	10.25378/janelia.7614368
AA0541	10.25378/janelia.7640162
AA0543	10.25378/janelia.7640168
AA0578	10.25378/janelia.7649858
AA0582	10.25378/janelia.7649873
AA0588	10.25378/janelia.7649933
AA0592	10.25378/janelia.7649948
AA0600	10.25378/janelia.7650029
AA0618	10.25378/janelia.7655729
AA0622	10.25378/janelia.7655753
AA0627	10.25378/janelia.7655771
AA0656	10.25378/janelia.7658219
AA0662	10.25378/janelia.7658243
AA0663	10.25378/janelia.7658246
AA0674	10.25378/janelia.7704212
AA0739	10.25378/janelia.7707326
AA0741	10.25378/janelia.7707338
AA0745	10.25378/janelia.7707359
AA0876	10.25378/janelia.7742576
AA0884	10.25378/janelia.7742789
AA0906	10.25378/janelia.7780859
**IT in MOs layer 2/3, Contralateral-Striatum-non-targeting**	
AA0014	10.25378/janelia.5521624
AA0116	10.25378/janelia.5526727
AA0118	10.25378/janelia.5526733
AA0237	10.25378/janelia.5527630
AA0238	10.25378/janelia.5527633
AA0241	10.25378/janelia.5527645
AA0418	10.25378/janelia.7614221
AA0426	10.25378/janelia.7614251
AA0446	10.25378/janelia.7614707
AA0471	10.25378/janelia.7615952
AA0474	10.25378/janelia.7615964
AA0738	10.25378/janelia.7707323
AA0782	10.25378/janelia.7739303
AA0793	10.25378/janelia.7739510
AA0802	10.25378/janelia.7739555
AA0803	10.25378/janelia.7739558
AA0915	10.25378/janelia.7780892
**IT in MOs layer 2/3, Contralateral-Striatum-targeting**	
AA0232	10.25378/janelia.5527609
AA0327	10.25378/janelia.7613498
AA0328	10.25378/janelia.7613507
AA0329	10.25378/janelia.7613525
AA0407	10.25378/janelia.7614158
AA0409	10.25378/janelia.7614182
AA0416	10.25378/janelia.7614215
AA0419	10.25378/janelia.7614227
AA0439	10.25378/janelia.7614329
AA0450	10.25378/janelia.7614965
AA0467	10.25378/janelia.7615901
AA0470	10.25378/janelia.7615940
AA0773	10.25378/janelia.7710113
AA0865	10.25378/janelia.7740089
AA0866	10.25378/janelia.7740092
AA0873	10.25378/janelia.7742567
AA0883	10.25378/janelia.7742786
AA0897	10.25378/janelia.7780811
**IT in MOs layer 5**	
AA0059	10.25378/janelia.5521780
AA0190	10.25378/janelia.5527474
AA0230	10.25378/janelia.5527603
AA0233	10.25378/janelia.5527612
AA0236	10.25378/janelia.5527621
AA0265	10.25378/janelia.5527738
AA0267	10.25378/janelia.5527747
AA0269	10.25378/janelia.5527753
AA0274	10.25378/janelia.5527774
AA0279	10.25378/janelia.5527792
AA0281	10.25378/janelia.5527798
AA0285	10.25378/janelia.5527810
AA0300	10.25378/janelia.5527855
AA0324	10.25378/janelia.7613486
AA0332	10.25378/janelia.7613684
AA0397	10.25378/janelia.7614113
AA0400	10.25378/janelia.7614122
AA0412	10.25378/janelia.7614200
AA0421	10.25378/janelia.7614233
AA0422	10.25378/janelia.7614236
AA0441	10.25378/janelia.7614356
AA0452	10.25378/janelia.7615274
AA0460	10.25378/janelia.7615835
AA0465	10.25378/janelia.7615889
AA0466	10.25378/janelia.7615895
AA0473	10.25378/janelia.7615961
AA0534	10.25378/janelia.7640063
AA0575	10.25378/janelia.7649846
AA0602	10.25378/janelia.7650038
AA0632	10.25378/janelia.7658054
AA0646	10.25378/janelia.7658126
AA0734	10.25378/janelia.7707296
AA0735	10.25378/janelia.7707302
AA0746	10.25378/janelia.7707365
AA0749	10.25378/janelia.7707374
AA0767	10.25378/janelia.7710077
AA0798	10.25378/janelia.7739537
AA0841	10.25378/janelia.7739909
AA0842	10.25378/janelia.7739915
AA0853	10.25378/janelia.7739954
AA0858	10.25378/janelia.7740017
AA0887	10.25378/janelia.7742807
AA0905	10.25378/janelia.7780856
**IT in MOs other layer or without layer description**	
AA0100	10.25378/janelia.5526679
AA0106	10.25378/janelia.5526697
AA0113	10.25378/janelia.5526718
AA0243	10.25378/janelia.5527651
AA0288	10.25378/janelia.5527819
AA0320	10.25378/janelia.7613465
AA0333	10.25378/janelia.7613693
AA0396	10.25378/janelia.7614107
AA0402	10.25378/janelia.7614128
AA0461	10.25378/janelia.7615838
AA0549	10.25378/janelia.7640201
AA0594	10.25378/janelia.7649966
AA0645	10.25378/janelia.7658114
AA0653	10.25378/janelia.7658153
AA0655	10.25378/janelia.7658210
AA0742	10.25378/janelia.7707347
AA0743	10.25378/janelia.7707350
AA0744	10.25378/janelia.7707356
AA0748	10.25378/janelia.7707371
AA0790	10.25378/janelia.7739384
AA0880	10.25378/janelia.7742777
AA0889	10.25378/janelia.7742816
AA0911	10.25378/janelia.7780874
**IT in other areas**	
AA0008	10.25378/janelia.5520451
AA0098	10.25378/janelia.5526673
AA0120	10.25378/janelia.5527234
AA0319	10.25378/janelia.7613459
AA0393	10.25378/janelia.7614098
AA0403	10.25378/janelia.7614131
AA0417	10.25378/janelia.7614218
AA0425	10.25378/janelia.7614245
AA0427	10.25378/janelia.7614254
AA0590	10.25378/janelia.7649942
AA0603	10.25378/janelia.7650041
AA0679	10.25378/janelia.7704230
AA0795	10.25378/janelia.7739519
AA0800	10.25378/janelia.7739543
AA0801	10.25378/janelia.7739549
AA0872	10.25378/janelia.7742564
**Corticostriatal neurons that we did not include into our analyses as IT or PT neurons**	
AA0096	10.25378/janelia.5526667
AA0105	10.25378/janelia.5526694
AA0231	10.25378/janelia.5527606
AA0284	10.25378/janelia.5527807
AA0395	10.25378/janelia.7614104
AA0413	10.25378/janelia.7614203
AA0445	10.25378/janelia.7614596
AA0457	10.25378/janelia.7615589
AA0469	10.25378/janelia.7615934
AA0492	10.25378/janelia.7616045
AA0636	10.25378/janelia.7658072
AA0641	10.25378/janelia.7658087
AA0642	10.25378/janelia.7658090
AA0644	10.25378/janelia.7658108
AA0647	10.25378/janelia.7658135
AA0668	10.25378/janelia.7658261
AA0671	10.25378/janelia.7704200
AA0747	10.25378/janelia.7707368
AA0765	10.25378/janelia.7710068
AA0784	10.25378/janelia.7739321
AA0807	10.25378/janelia.7739642
AA0840	10.25378/janelia.7739903
AA0870	10.25378/janelia.7742552
AA0900	10.25378/janelia.7780826
AA0914	10.25378/janelia.7780883
AA0919	10.25378/janelia.7780907

*The source of the data is the MouseLight project at Janelia (http://ml-neuronbrowser.janelia.org/).*

Next we searched entities of IT type CS neurons. A potential strategy was to search neurons having axons in the contra-Str, since previous studies have described that only IT neurons, but not PT neurons, can project to the contra-Str (Miller, 1975; Catsman-Berrevoets et al., 1980; Wilson, 1986). However, because IT neurons do not necessarily project to the contra-Str and also because practically we could not find a way to specify the contra-Str in the Neuron Browser, we took a different strategy. Specifically, we conducted a separate search of the Neuron Browser by setting only filters (1) and (2) mentioned above, omitting filter (3) (axon in pons), and manually excluded entities that were also found in the search with filter (3) so as to obtain candidates of IT neurons. This yielded 187 entities of neocortical neurons with all the structures; neurons with soma not located in the neocortex (but in the hippocampus or hippocampal formation in most cases) were also manually excluded.

In order to check if these 187 neurons satisfy the definition of IT neurons, i.e., axon projections only within the telencephalon, we examined JSON files of these entities downloaded from the MouseLight database and checked if axon exists in “allenIds” that are considered to be outside of the telencephalon (but omitting some “allenIds” corresponding to tract, bundle, or ventricle, parts of which could potentially be IT, such as the corticospinal tract). As a result, 26 (out of 187) entities were found to have axon in non-IT regions (Table 2). Of these 26 entities, 11 are neurons with a substantial portion of axons (>10% of axon entities in JSON file) in non-IT regions, such as thalamus, midbrain, or hypothalamus, and would thus be inappropriate to be labeled as IT neurons. Among them, AA0919 and AA0644 having soma in layer 5 could potentially be PT neurons, although we did not include them into our analyses as PT neurons. Other than these and a neuron having soma in layer 2/3, 8 out of the 11 neurons having soma in layer 6a and targeting thalamus would be corticothalamic neurons. Layer 6 corticothalamic neurons are distinct from PT neurons, and they together constitute extratelencephalic neurons (Baker et al., 2018). Layer 6 striatum-targeting corticothalamic neurons can thus be regarded as a third type of CS neurons (i.e., other than IT and PT neurons), but here we did not further analyze them. The remaining 15 entities are neurons whose non-IT axon projections are limited (<2.2% of axon entities in JSON file), and it might be good to classify them together with properly IT neurons, although we did not do so.

**TABLE 2:** List of entities of corticostriatal neurons in the MouseLight database that we did not include into our analyses as IT or PT neurons.

**Proportion of non-IT axons**	**ID**	**Area**	**Layer**	**Major non-IT projecting region(s)**
0.95	AA0641	MOp	6a	thalamus, midbrain reticular nucleus
0.90	AA0642	MOp	6a	thalamus, midbrain reticular nucleus
0.85	AA0784	MOs	6a	thalamus
0.84	AA0647	MOp	6a	thalamus
0.74	AA0457	MOs	6a	thalamus
0.71	AA0231	MOs	6a	thalamus, nucleus of reunions
0.52	AA0105	MOs	6a	thalamus
0.33	AA0919	Dorsal auditory area	5	midbrain reticular nucleus, thalamus, inferior colliculus, midbrain
0.25	AA0807	Anterior cingulate area, dorsal part	2/3	midbrain reticular nucleus, hypothalamus, ventral tegmental area, superior colliculus, zona incerta
0.14	AA0644	MOp	5	thalamus, posterior hypothalamic nucleus
0.14	AA0747	MOs	6a	hypothalamus, cerebral peduncle, lateral hypothalamic area, SNc, mammillary peduncle
0.02	AA0469	MOs	1	SNr, lateral lemniscus, cerebral peduncle
0.02	AA0914	MOs	2/3	lateral hypothalamic area, hypothalamus
0.02	AA0671	MOs	2/3	lateral hypothalamic area, hypothalamus, parasubthalamic nucleus, midbrain, hypothalamic lateral zone
0.01	AA0636	Prelimbic area	2/3	thalamus, hypothalamic lateral zone, lateral hypothalamic area, hypothalamus
0.01	AA0765	Anterior cingulate area, dorsal part	2/3	lateral hypothalamic area, hypothalamus, hypothalamic lateral zone, interbrain, brain stem
0.01	AA0492	Primary visual area	6a	inferior colliculus, midbrain, superior colliculus, brain stem
0.01	AA0870	Prelimbic area	2/3	lateral hypothalamic area, brain stem, hypothalamus, hypothalamic lateral zone
0.01	AA0900	MOs	6a	subthalamic nucleus, hypothalamic lateral zone
0.00	AA0096	Anterior cingulate area dorsal part	2/3	midbrain, inferior colliculus, superior colliculus, brain stem
0.00	AA0413	MOs	2/3	hypothalamus, interbrain, brain stem
0.00	AA0284	MOs	2/3	lateral hypothalamic area
0.00	AA0668	MOs	no description	lateral hypothalamic area, hypothalamus
0.00	AA0445	MOs	5	lateral hypothalamic area, hypothalamus
0.00	AA0395	MOs	2/3	lateral preoptic area
0.00	AA0840	MOs	5	stria medullaris

**FIGURE 1:**
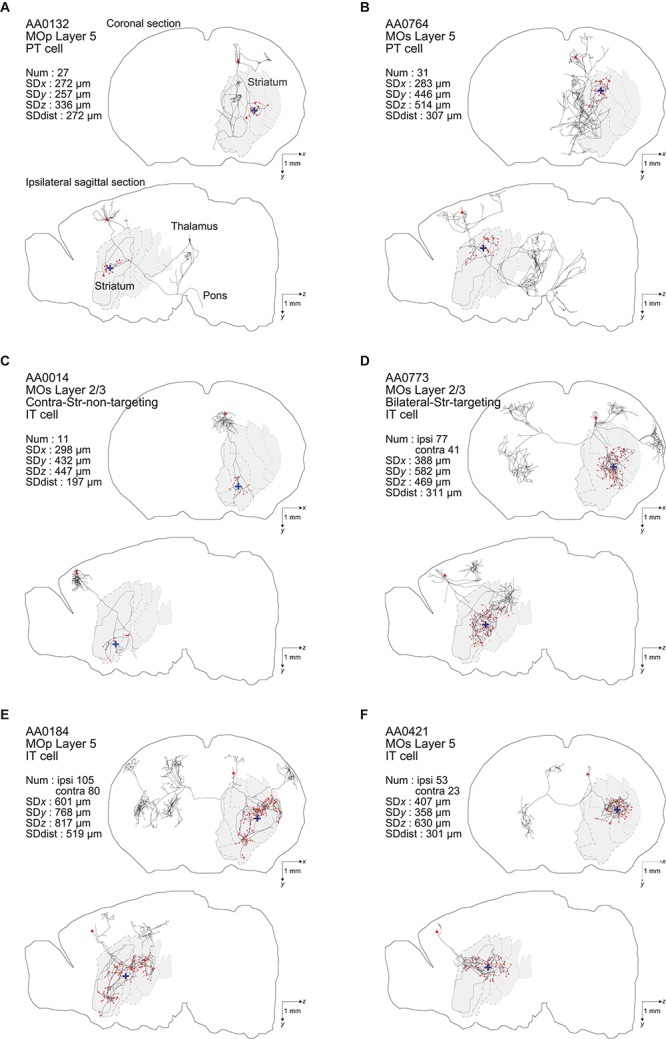
Examples of axonal arborizations of PT and IT neurons, drawn by using the data downloaded from the MouseLight database (http://ml-neuronbrowser.janelia.org/). Coronal (*x*-*y* plane) and sagittal (*y*-*z* plane) sections are shown for individual cells. The red points indicate the axonal end-points ipsilateral to the soma, and the blue cross indicates the center of those end-points (i.e., the point whose coordinates are the means of the *x*, *y*, and *z* coordinates of individual end-points). The small red triangle indicates the soma. The text in the top-left of each panel describes the following information: ID number in the MouseLight database; Cortical area of soma [primary motor area (MOp) or secondary motor area (MOs)]; Layer of soma, Neuron type (PT or IT); Num: number of axonal end-points in the ipsilateral striatum **(A–C)** or in the bilateral striatum **(D–F)**; SD*x*, SD*y*, SD*z*: the standard deviation (SD) of *x*, *y*, and *z* coordinates of the ipsilateral intra-striatal end-points (in [μm]); and SD dist: SD of the distances of ipsilateral intra-striatal end-points from the center of those end-points (in [μm]). The contours of striatum were referred from Allen 3-D annotation (Kuan et al., 2015) and filled in gray.

In the end, we analyzed 161 (= 187–26) entities as IT neurons. Of these, 44 neurons are in the primary motor area (MOp) [3 in layer 1 (according to the annotation in the MouseLight database), 6 in layer 2/3, 23 in layer 5, and 12 in layer 6a], 101 neurons are in the secondary motor area (MOs) (4 in layer 1, 35 in layer 2/3, 43 in layer 5, 17 in layer 6a, and 2 without description of layer), and the remaining 16 neurons are in other neocortical areas. Also, we analyzed 33 entities as PT neurons (all with soma in layer 5 as mentioned above). Of these, 8, 16, and 9 neurons are in the MOp, MOs, and other neocortical areas, respectively. Figure 1 shows the axon morphology of examples of PT and IT neurons. (Note: although we did not record the numbers of search results at our original searches in the MouseLight database, we later realized that additionally specifying “Fundus of striatum” and “Olfactory tubercle” for filter (2), or specifying “Striatum” only for filter (2) and “Pons” only for filter (3), does not change the number of search results.)

### Analysis of Axonal End-Points in the Striatum

We analyzed JSON files of the identified IT and PT neurons downloaded from the MouseLight database to identify axonal end-points in the striatum, i.e., axon samples (“structureIdentifier”: 2) that have “allenIds” of 477 (Striatum), 485 (Striatum dorsal region), 493 (Striatum ventral region), 672 (Caudoputamen), 56 (Nucleus accumbens), 754 (Olfactory tubercle), or 998 (Fundus of striatum) and are not a parent of other axon samples (i.e., whose “sampleNumber” does not appear as “parentNumber” of other axon samples); later we re-identified axonal end-points in the striatum with additional allenIds, 481 (Islands of Calleja), 489 (Major island of Calleja), 144 (Olfactory tubercle, layers 1–3), 458 (Olfactory tubercle, molecular layer), 465 (Olfactory tubercle, pyramidal layer), and 473 (Olfactory tubercle, polymorph layer), also included in the identification process, but no extra end-point was identified. We classified the identified axonal end-points into either ipsi- or contra-lateral axonal end-points by examining whether the *x* coordinate (right-left position) is at the same side as the soma with respect to *x* = 5500, which appeared to be near the midline when we plotted the distribution of *x* coordinates of striatal axonal end-points in an example neuron. Later we realized that *x* = 5700 would actually be around the midline, but we have confirmed that the number of ipsi/contralateral intra-striatal axonal end-points and their coordinates for all the IT and PT neurons do not change when *x* = 5700, instead of *x* = 5500, is used in the extraction.

We did analyses with MATLAB (MathWorks Inc.), using custom-made codes and the codes for statistical analyses formerly in http://rnpsychology.org/ (by Ryosuke Niimi), and R (https://www.r-project.org/). Rounding errors were introduced when data were moved from MATLAB to CSV files using csvwrite.m for analyses using R. For χ^2^ test, we calculated φ = sqrt(χ^2^/(*N*_1_+*N*_2_)), where sqrt denotes square root, χ^2^ is the chi-square statistic, and *N*_1_ and *N*_2_ are the numbers of the samples. For Welch’s *t*-test, we calculated *d* = |μ_1_−μ_2_|/sqrt((((*N*_1_−1)*s*_1_^2^)+((*N*_2_−1)*s*_2_^2^))/(*N*_1_+*N*_2_−2)), where μ_1_ and μ_2_ or *s*_1_ and *s*_2_ are the means or standard deviations of the samples.

## Results

### Number of Intra-Striatal Axonal End-Points

**FIGURE 2:**
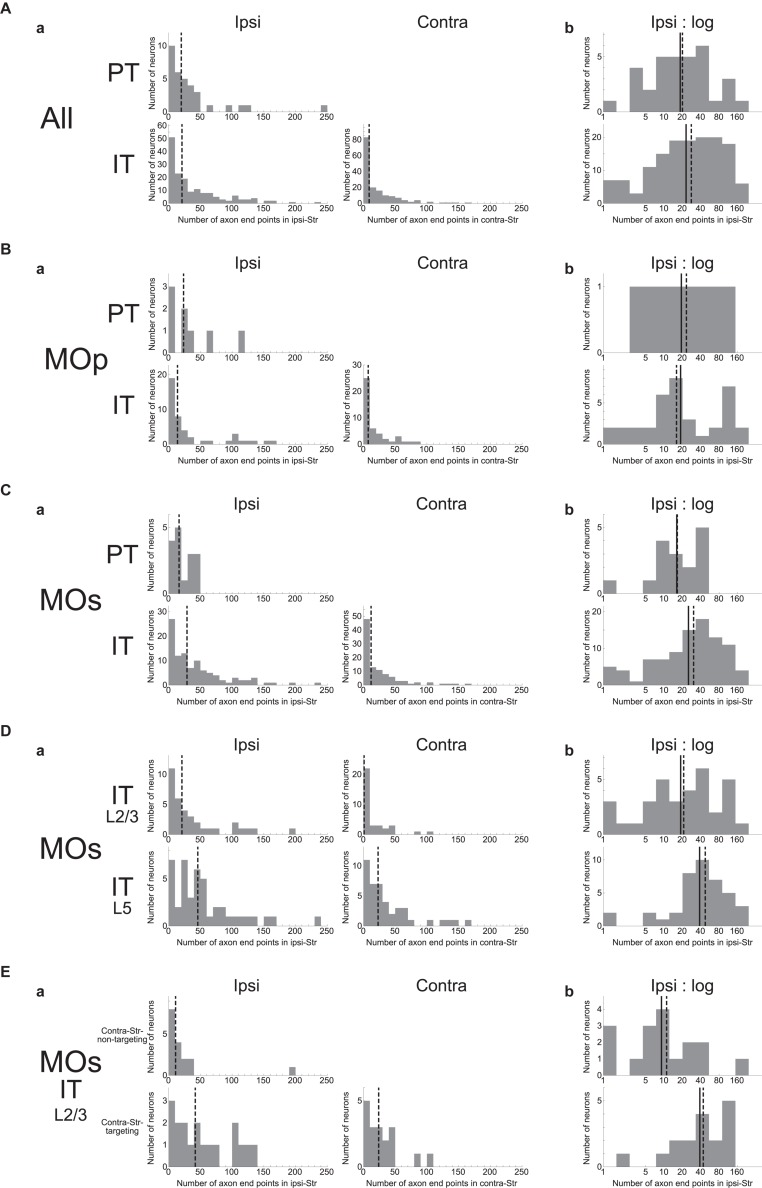
Distributions of the number of intra-striatal axonal end-points. **(A)**; **(a)** Distributions for all the striatum-targeting PT and IT neurons (33 and 161 neurons, respectively) that we identified in the MouseLight database. IT neurons having no ipsi-(16) or contra-(42) lateral axonal end-point are included in the histograms. The dashed lines indicate the medians. **(b)** Distributions of the logarithm of the number of ipsilateral intra-striatal axonal end-points for the striatum-targeting 33 PT and 145 IT neurons that have at least one ipsilateral end-point; i.e., IT neurons having no ipsilateral axonal end-point (16 neurons) are excluded from the histogram [the same is applied also to the following histograms in **(b)**]. The solid lines and the dashed lines indicate the means and the medians, respectively; the same is applied also to the following figures. **(B,C)** Results for the neurons whose somata are located in the primary motor area [**(a)** 8 PT and 44 IT neurons (including 7 w/o ipsi-point and 10 w/o contra-point), **(b)** 8 PT and 37 IT neurons] **(B)** or the secondary motor area (MOs) [**(a)** 16 PT and 101 IT neurons (including 7 w/o ipsi-point and 27 w/o contra-point), **(b)** 16 PT and 94 IT neurons] **(C)**. **(D)** Results for MOs layer (L) 2/3 IT neurons [**(a)** 35 neurons including 1 w/o ipsi-point and 17 w/o contra-point, **(b)** 34 neurons] and MOs layer 5 IT neurons [**(a)** 43 neurons including 3 w/o ipsi-point and 4 w/o contra-point, **(b)** 40 neurons]. **(E)** Results for MOs layer 2/3 IT neurons that do not target contralateral striatum (contra-Str) (17 neurons) and those that target contra-Str [**(a)** 18 neurons including 1 w/o ipsi-point, **(b)** 17 neurons].

We examined how the number of intra-striatal axonal end-points is distributed across neuron types as well as across individual neurons. As shown in Figure 2Aa, the number turned out to be widely distributed across individual neurons in both PT and IT neural populations and for both ipsi- and contra-lateral axonal end-points in the case of IT neurons (no PT neuron has axonal end-point in the contra-lateral striatum, consistent with previous studies). In all the cases, the distributions are roughly monotonically decreasing, i.e., neurons with ≤20 axonal end-points are most frequent, while there are also neuron(s) having ≥100 axonal end-points. Comparing the ipsi- and contra-lateral axonal end-points in IT neurons, there tend to be more ipsilateral end-points than contra-lateral end-points: ipsi-points outnumber contra-points in 122 (out of 161) neurons, including 42 neurons without contra-points, whereas contra-points outnumber ipsi-points in 38 neurons, including 16 neurons without ipsi-points (the remaining 1 neuron has the same numbers of ipsi- and contra-points).

Comparing the ipsilateral axonal end-points between PT and IT neurons, the proportion of neurons having more than 50 ipsi-points within those having at least one ipsi-point is somewhat larger in IT neurons (48/145 = 0.33) than in PT neurons (5/33 = 0.15) (χ^2^ test, *p* = 0.042, φ = 0.15). Nonetheless, the intra-neuron-type variabilities across individual neurons looks more prominent than the inter-neuron type variability. Indeed, slightly changing the abovementioned threshold number of end-points (i.e., 50) makes the difference between PT and IT neurons non-significant. Moreover, the distributions of the logarithm of the number of ipsilateral axonal end-points, excluding the neurons having no ipsilateral end-point (this is also applied to all the following analyses dealing with the logarithm of the number of end-points so as to avoid “log 0”), are considerably overlapped between PT and IT neurons (Figure 2Ab) (Welch’s *t*-test, *p* = 0.36). Figures 2B,C are the results of analyses limited to neurons in the MOp or MOs, respectively, showing similar tendencies to the results of analyses including all the neurons.

We also analyzed if MOs IT neurons in layer 2/3 and those in layer 5 differ in the number of intra-striatal axonal end-points (Figure 2D). It turned out that whereas most layer 5 MOs IT neurons (39 out of 43) have at least one axonal end-point in the contra-Str and 36 of them target bilateral striatum, only about a half of layer 2/3 MOs IT neurons (18 out of 35) have contralateral striatal end-point(s). There is also a trend that the proportion of neurons having more than 50 ipsilateral end-points within those having at least one ipsi-point tends to be larger in layer 5 MOs IT neurons (18/40 = 0.45) than in layer 2/3 neurons (9/34 = 0.26) (χ^2^ test, *p* = 0.099, φ = 0.19). Moreover, the distributions of the logarithm of the number of ipsilateral axonal end-points, excluding the neurons having no ipsilateral end-point, differ between layer 2/3 and layer 5 MOs IT neurons (Welch’s *t*-test, *p* = 0.024, *d* = 0.55), with the layer 5 neurons on average having a larger number of ipsilateral end-points than the layer 2/3 neurons as apparent in Figure 2Db. The distributions also differ between MOs layer 5 IT neurons and MOs PT neurons (*p* = 0.0097, *d* = 0.76) but not between MOs layer 2/3 IT neurons and MOs PT neurons (*p* = 0.68).

As mentioned above, whereas most of MOs layer 5 IT neurons (39/43) project to contra-Str and 36 of them target bilateral striatum, MOs layer 2/3 IT neurons are almost bisected into those targeting contra-Str (18/35) and those not targeting (17/35). Except for a contra-Str-targeting layer 2/3 neurons that does not target ipsilateral striatum, the remaining bilateral-Str-targeting layer 2/3 neurons (17) on average have a larger number of ipsilateral end-points than the contra-Str-non-targeting layer 2/3 neurons (Figure 2E), with the distributions of the logarithm of the number of end-points significantly different (Welch’s *t*-test, *p* = 0.0022, *d* = 1.15). The distribution for bilateral-Str-targeting MOs layer 2/3 IT neurons does not differ from that for MOs layer 5 IT neurons (*p* = 0.97) but differs from that for MOs PT neurons (*p* = 0.024, *d* = 0.82). On the contrary, the distribution for contra-Str-non-targeting MOs layer 2/3 IT neurons differs from that for MOs layer 5 IT neurons (*p* = 0.0010, *d* = 1.16) but hardly differs from that for MOs PT neurons (*p* = 0.19).

There is a note regarding the layer classification of MOs IT neurons. As mentioned above, there are 18 contra-Str-targeting layer 2/3 neurons in the dataset that we analyzed. They amount to 24% of 74 contra-Str-targeting MOs IT neurons. This proportion appears to be higher than the proportion in other study in rat (Hirai et al., 2012; Figure 1B of this cited paper). This difference may come from the differences in species and other factors. However, given that the cortex around MOs shows a gyrus-like high convexity and the superficial layers become flattened (Hooks et al., 2011), there might be a possibility that some of the layer 2/3 neurons that we analyzed could in fact be neurons in the upper part of layer 5 (layer 5a).

### Spatial Distribution of Intra-Striatal Axonal End-Points

**FIGURE 3:**
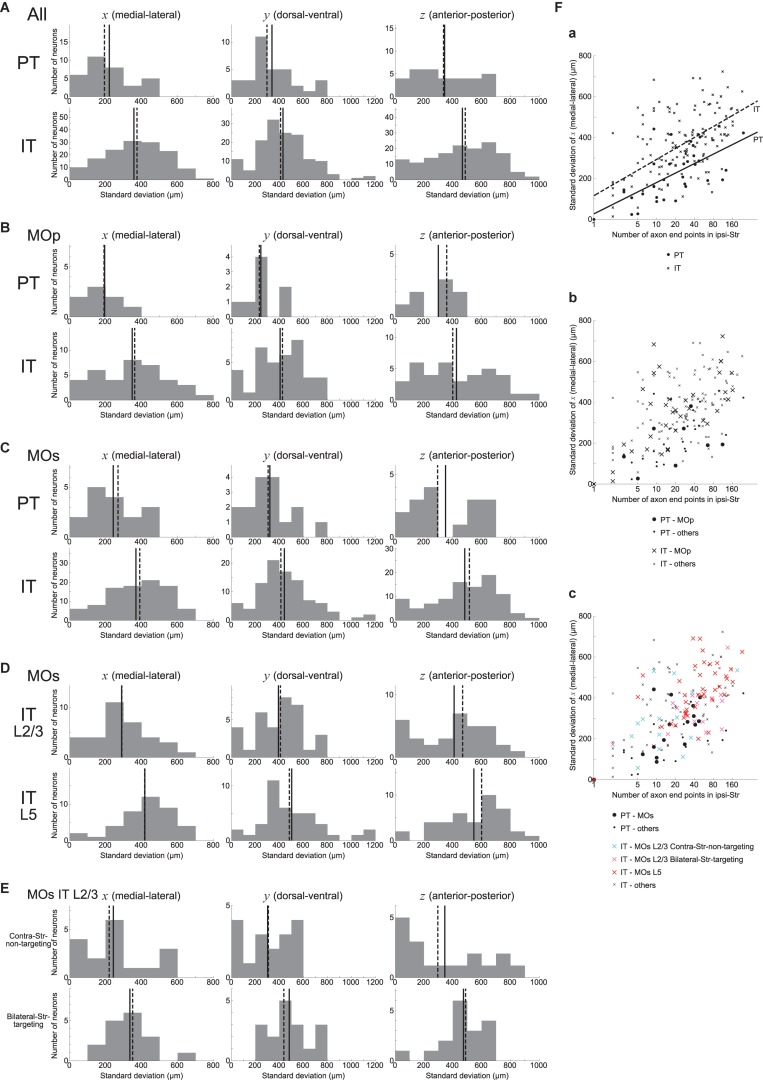
Distributions of the standard deviation (SD) of the spatial coordinates of ipsilateral intra-striatal axonal end-points. **(A)** The left, middle, and right panels show the distributions of the SD of *x*, *y*, and *z* coordinates (corresponding to the medial-lateral (or right-left), dorsal-ventral (or top-bottom), and anterior-posterior directions) of ipsilateral axonal end-points, respectively, for the striatum-targeting 33 PT (top panels) and 145 IT (bottom panels) neurons that have at least one ipsilateral end-point; i.e., IT neurons having no ipsilateral axonal end-point (16 neurons) are excluded from the histograms (the same is applied also to the following histograms). **(B,C)** Results for the neurons whose somata are located in the primary motor area (MOp) [8 PT and 37 IT neurons (excluding 7 IT neurons w/o ipsi-point)] **(B)** or the secondary motor area (MOs) [16 PT and 94 IT neurons (excluding 7 IT neurons w/o ipsi-point)] **(C)**. **(D)** Results for MOs layer 2/3 IT neurons (34 neurons, excluding 1 w/o ipsi-point) and MOs layer 5 IT neurons (40 neurons, excluding 3 w/o ipsi-point). **(E)** Results for MOs layer 2/3 IT neurons that do not target contra-Str (17 neurons) and those targeting bilateral-Str (17 neurons). **(F)** Relationship between the logarithm of the number of ipsilateral axonal end-points (horizontal axis) and the SD of *x* coordinates of the end-points (vertical axis). **(a)** The circles and crosses indicate all the striatum-targeting PT and IT neurons that have at least one ipsilateral end-point, respectively. There is a positive correlation between the two variables in both PT neurons (*p* = 6.4 × 10^–5^, *r* = 0.64; *p* = 3.9 × 10^–4^, *r* = 0.59 when excluding a neuron with only one ipsilateral end point) and IT neurons (*p* = 7.5 × 10^–17^, *r* = 0.62; *p* = 9.1 × 10^–10^, *r* = 0.49 w/o neurons with one ipsi-point). The solid and dashed lines indicate the fitted lines of linear regression for PT and IT neurons, respectively [PT: intercept 27.0 (*p* = 0.56), slope 66.7 (*p* = 6.4 × 10^–5^); IT: intercept 115.8 (*p* = 5.2 × 10^–5^), slope 77.2 (*p* < 2 × 10^–16^)]. **(b)** The neurons in MOp are indicated by large symbols. Positive correlation between the two variables exists for MOp IT neurons (*p* = 2.9 × 10^–6^, *r* = 0.69; *p* = 1.6 × 10^–4^, *r* = 0.60 w/o neurons with one ipsi-point), but not for MOp PT neurons (*p* = 0.31; every neuron has>1 ipsi-points). **(c)** MOs PT neurons, MOs layer 2/3 IT contra-Str-non-targeting neurons, MOs layer 2/3 IT bilateral-Str-targeting neurons, and MOs layer 5 IT neurons are indicated by black large circles, light blue large crosses, pink large crosses, and red large crosses, respectively. Positive correlation between the two variables exists for MOs PT neurons (*p* = 0.0059, *r* = 0.66; *p* = 0.065, *r* = 0.49 w/o a neuron with one ipsi-point) and MOs entire IT neurons (*p* = 9.4 × 10^–11^, *r* = 0.61; *p* = 2.5 × 10^–5^, *r* = 0.43 w/o neurons with one ipsi-point).

We also examined how the ipsilateral intra-striatal axonal end-points are spatially distributed. Specifically, we calculated the standard deviation (SD) of *x*, *y*, and *z* coordinates (corresponding to the medial-lateral (or right-left), dorsal-ventral (or top-bottom), and anterior-posterior directions, respectively) of the ipsilateral end-point(s) for each neuron, excluding the neurons having no ipsilateral end-point (this is also applied to all the following analyses dealing with the SD of the coordinates). As shown in Figure 3A, the SD is distributed from 0 to several hundreds or up to 1200 μm. Comparing PT and IT neurons, axonal end-points of IT neurons on average have larger SD of the coordinates than those of PT neurons for all the three coordinates, with the most prominent difference for *x* coordinate (Figure 3A) (Welch’s *t*-test, *x*: *p* = 2.5 × 10^–6^, *d* = 0.85; *y*: *p* = 0.019, *d* = 0.42; *z*: *p* = 0.0024, *d* = 0.55). The same tendencies also appear when analysis is limited to neurons in MOp or MOs (Figures 3B,C) (MOp, *x*: *p* = 0.0076, *d* = 0.86; *y*: *p* = 0.014, *d* = 0.81; *z*: *p* = 0.10, *d* = 0.53; and MOs, *x*: *p* = 0.0020, *d* = 0.79; *y*: *p* = 0.028, *d* = 0.54; *z*: *p* = 0.029, *d* = 0.59). It is therefore suggested that the ipsilateral intra-striatal axonal end-points of IT neurons are on average more spatially extended than those of PT neurons, especially for the medial-lateral direction.

We also analyzed if MOs IT neurons in layer 2/3 and those in layer 5 differ in the spatial distribution of ipsilateral intra-striatal axonal end-points. As shown in Figure 3D, it turned out that the SD of the spatial coordinates of end-points is on average larger for MOs layer 5 IT neurons than for MOs layer 2/3 IT neurons in all the three directions, with the most prominent difference in the medial-lateral (*x*) direction (Welch’s *t*-test, *x*: *p* = 7.8 × 10^–4^, *d* = 0.82; *y*: *p* = 0.045, *d* = 0.47; *z*: *p* = 0.013, *d* = 0.60). The distributions differ also between MOs layer 5 IT neurons and MOs PT neurons (*x*: *p* = 1.3 × 10^–4^, *d* = 1.18; *y*: *p* = 0.0054, *d* = 0.75; *z*: *p* = 0.0036, *d* = 0.91) but not between MOs layer 2/3 IT neurons and MOs PT neurons (*x*: *p* = 0.27; *y*: *p* = 0.22; *z*: *p* = 0.37).

As mentioned above, about a half of MOs layer 2/3 IT neurons (17/35) target bilateral striatum. The bilateral-Str-targeting neurons tend to have wider spatial distributions of ipsilateral end-points than contra-Str-non-targeting neurons (Figure 3E) (Welch’s *t*-test, *x*: *p* = 0.092, *d* = 0.60; *y*: *p* = 0.0074, *d* = 0.99; *z*: *p* = 0.12, *d* = 0.55). The spatial distributions for the contra-Str-non-targeting neurons are narrower than layer 5 IT neurons (*x*: *p* = 0.0019, *d* = 1.06; *y*: *p* = 0.0036, *d* = 0.81; *z*: *p* = 0.019, *d* = 0.83), and comparable to PT neurons (*x*: *p* = 0.99; *y*: *p* = 0.79; *z*: *p* = 0.96). By contrast, the spatial distributions for the bilateral-Str-targeting neurons are narrower in *x*- but comparable in *y*- and *z*- coordinates compared to layer 5 IT neurons (*x*: *p* = 0.033, *d* = 0.57; *y*: *p* = 0.72; *z*: *p* = 0.16), and wider than PT neurons (*x*: *p* = 0.039, *d* = 0.75; *y*: *p* = 0.011, *d* = 0.95; *z*: *p* = 0.059, *d* = 0.69).

We also examined the relationship between the logarithm of the number of ipsilateral end-points and the SD of their *x* coordinates. As shown in Figure 3Fa, these two variables are positively correlated in both PT and IT neurons (see the legend for details). Moreover, results of linear regression of the SD of *x* coordinates against the logarithm of the number of end-points (PT: solid line; IT: dashed line; see the legend for details) indicate that IT neurons tend to have larger SD of *x* coordinates of end-points than PT neurons with comparable number of end-points. Also, the scatter plot distinguishing subpopulations of MOs IT and PT neurons (Figure 3Fc) indicates that layer 2/3 contra-Str-non-targeting IT neurons (light-blue crosses) are distinct from layer 2/3 bilateral-Str-targeting (pink crosses) or layer 5 (red crosses) IT neurons and closer to PT neurons (black circles), in line with the results described in the previous paragraphs.

**FIGURE 4:**
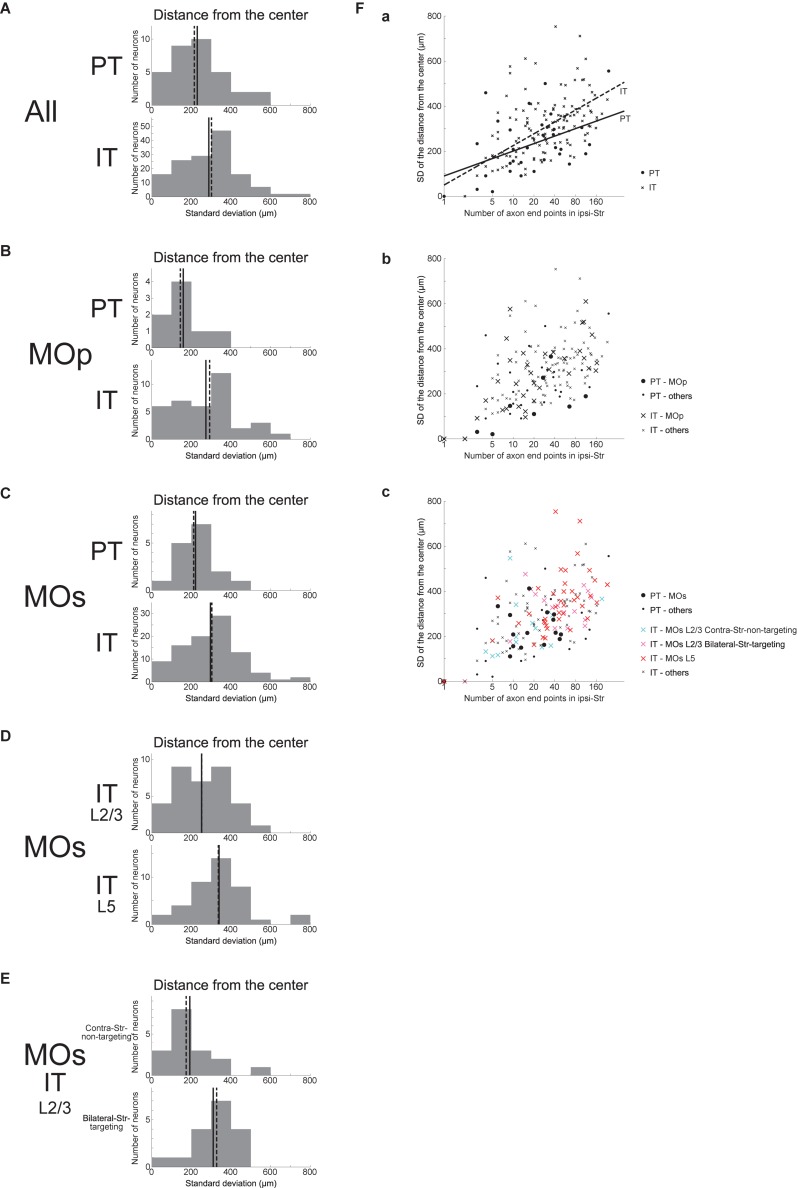
Distributions of the SD of the distances between the individual ipsilateral intra-striatal axonal end-points and the center of the end-points (i.e., the point whose coordinates are the means of the *x*, *y*, and *z* coordinates of individual end-points). **(A)** The top and bottom panels show the results for the striatum-targeting PT and IT neurons that have at least one ipsilateral end-point, respectively. IT neurons having no ipsilateral axonal end-point are excluded from the histograms, and the numbers of neurons included in, and excluded from, the histograms are the same as those in Figure 3 (the same is applied also to the following histograms). **(B,C)** Results for the neurons whose somata are located in the primary motor area (MOp) **(B)** or the secondary motor area (MOs) **(C)**. **(D)** Results for MOs layer 2/3 IT neurons and MOs layer 5 IT neurons. **(E)** Results for MOs layer 2/3 IT neurons that do not target contra-Str and those that target bilateral-Str. **(F)** Relationship between the logarithm of the number of ipsilateral axonal end-points (horizontal axis) and the SD of the distances of end-points from the center of end-points (vertical axis). **(a)** The circles and crosses indicate all the striatum-targeting PT and IT neurons that have at least one ipsilateral end-point, respectively. There is a positive correlation between the two variables in both PT neurons (*p* = 0.0098, *r* = 0.44; *p* = 0.042, *r* = 0.36 when excluding a neuron with only one ipsilateral end point) and IT neurons (*p* = 6.4 × 10^–20^, *r* = 0.67; *p* = 1.8 × 10^–13^, *r* = 0.57 w/o neurons with one ipsi-point). The solid and dashed lines indicate the resulting fitted lines of linear regression for PT and IT neurons, respectively [PT: intercept 90.3 (*p* = 0.11), slope 48.1 (*p* = 0.0098); IT: intercept 50.0 (*p* = 0.041), slope 76.1 (*p* < 2 × 10^–16^)]. **(b)** The neurons in MOp are indicated by large symbols. Positive correlation between the two variables exists for MOp IT neurons (*p* = 6.8 × 10^–7^, *r* = 0.71; *p* = 2.8 × 10^–5^, *r* = 0.65 w/o neurons with one ipsi-point), and tends to exist for MOp PT neurons (*p* = 0.10, *r* = 0.62; every neuron has >1 ipsi-points). **(c)** MOs PT neurons, MOs layer 2/3 IT contra-Str-non-targeting neurons, MOs layer 2/3 IT bilateral-Str-targeting neurons, and MOs layer 5 IT neurons are indicated by black large circles, light blue large crosses, pink large crosses, and red large crosses, respectively. Positive correlation between the two variables exists for MOs entire IT neurons (*p* = 1.6 × 10^–12^, *r* = 0.65; *p* = 8.4 × 10^–8^, *r* = 0.53 w/o neurons with one ipsi-point), but hardly exists for MOs PT neurons (*p* = 0.083, *r* = 0.45; *p* = 0.95, *r* = 0.017 w/o a neuron with one ipsi-point).

As a different measure of spatial extent of axonal end-points that unifies the three (i.e., *x*, *y*, and *z*) directions, we calculated the SD of the distances between the individual ipsilateral intra-striatal axonal end-points and the center of these end-points (i.e., the point whose coordinates are the means of the *x*, *y*, and *z* coordinates of individual end-points) for each neuron, excluding the neurons having no ipsilateral end-point (this is also applied to all the following analyses dealing with the SD of the distances). This SD of the distances turned out to be on average larger for IT neurons than for PT neurons (Figure 4A) (Welch’s *t*-test, *p* = 0.032, *d* = 0.39), confirming that IT axonal end-points are spatially more extended than PT end-points. This relation also holds for MOp neurons only (Figure 4B) (*p* = 0.036, *d* = 0.73) or MOs neurons only (Figure 4C) (*p* = 0.015, *d* = 0.51). Distinguishing the layers of MOs IT neurons (Figure 4D), the SD of the distances for layer 5 neurons is on average larger than that for layer 2/3 neurons (*p* = 0.011, *d* = 0.61), and the former is also larger than the value for MOs PT neurons (*p* = 0.0012, *d* = 0.86) whereas the latter is comparable to the value for MOs PT neurons (*p* = 0.38). Further distinguishing bilateral-Str-targeting and contra-Str-non-targeting MOs layer 2/3 IT neurons (Figure 4E), the SD of the distances for bilateral-Str-targeting neurons is larger than that for contra-Str-non-targeting neurons (*p* = 0.011, *d* = 0.92), and the former is also larger than the value for MOs PT neurons (*p* = 0.021, *d* = 0.85) and comparable to MOs layer 5 IT neurons (*p* = 0.43) whereas the latter is comparable to the value for MOs PT neurons (*p* = 0.50) and smaller than the value for MOs layer 5 IT neurons (*p* = 0.0013, *d* = 1.00).

We also examined the relationship between the logarithm of the number of ipsilateral end-points and the SD of the distances of these end-points from their center (Figure 4F). As shown in Figure 4Fa, these two variables are positively correlated in both PT and IT neurons (see the legend for details). Results of linear regression of the SD of the distances against the logarithm of the number of end-points (PT: solid line; IT: dashed line; see the legend for details) indicate a somewhat steeper slope for IT neurons, but the difference between the PT and IT neurons is not drastic compared with the results of linear regression of the SD of *x* coordinates against the logarithm of the number of end-points (Figure 3Fa). Meanwhile, the scatter plot distinguishing subpopulations of MOs IT and PT neurons (Figure 4Fc) indicates that layer 2/3 contra-Str-non-targeting IT neurons (light-blue crosses) are distinct from layer 2/3 bilateral-Str-targeting (pink crosses) or layer 5 (red crosses) IT neurons and closer to PT neurons (black circles), in line with the results described in the previous paragraph and similarly to the results of linear regression of the SD of *x* coordinates against the logarithm of the number of end-points (Figure 3Fa).

## Discussion

The present work addressed the long-standing issue, whether IT CS axons are morphologically more extensive than PT axons, by taking advantage of the recently developed public database of neuron morphology, in which we identified 33 and 161 striatum-targeting PT and IT neurons, respectively. Counting the number of intra-striatal axonal end-points, we have shown that there exists a large variety in the number of end-points across neurons in both neuron types. This variety seems in line with the suggested heterogeneity and existence of sub-types within each of PT and IT populations (Economo et al., 2018; Winnubst et al., 2019). More specifically, we found that, among MOs IT neurons, layer 5 neurons have a larger number of ipsilateral end-points than layer 2/3 neurons, and also bilateral-Str-targeting layer 2/3 neurons have a larger number of ipsilateral end-points than contra-Str-non-targeting layer 2/3 neurons. In contrast to these within-neuron-type differences, the entire IT and PT neurons turned out to be not drastically different in the number of ipsilateral end-points. This may be consistent with the previous study (Zheng and Wilson, 2002), which concluded that the once suggested difference between IT and PT axon morphology was spurious. Nonetheless, with the data of much increased number of neurons in the MouseLight database, we have shown that the proportion of neurons having more than 50 ipsilateral intra-striatal axonal end-points, within neurons having at least one ipsi-point, is larger in IT neurons than in PT neurons, although the difference is relatively small and slightly changing the threshold number of end-points (i.e., 50) makes the difference non-significant. Moreover, in MOs, layer 5, and bilateral-Str-targeting layer 2/3 IT neurons, but not contra-Str-non-targeting layer 2/3 IT neurons, have a larger number of ipsilateral end-points than PT neurons.

**FIGURE 5:**
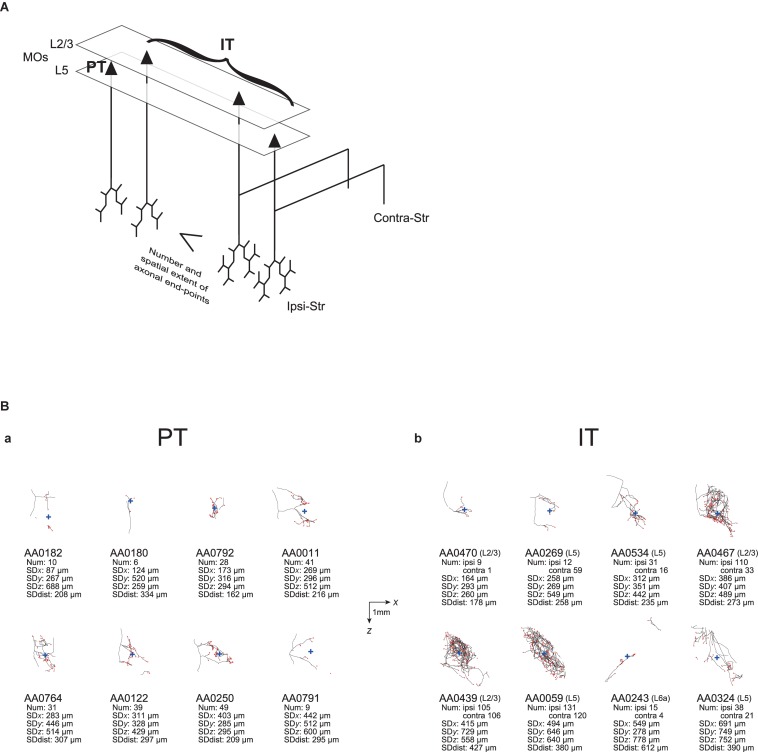
Summary of the properties of ipsilateral striatal axonal end-points of MOs PT and IT neurons, and examples of axonal arborizations. **(A)** Summary of the properties of ipsilateral striatal axonal end-points of MOs PT neurons and IT subpopulations. Compared with PT neurons and contralateral striatum-non-targeting layer 2/3 IT neurons, bilateral striatum-targeting layer 2/3 IT neurons and layer 5 IT neurons on average have a larger number, and a wider spatial extent, of ipsilateral axonal end-points. Notably, there are large varieties within PT and each of IT subpopulations, although they are not drawn in this figure. Also note that minorities of neurons (layer 2/3 IT neuron targeting contra-Str only and layer 5 IT neurons targeting ipsi-Str or contra-Str only) are not drawn here, and also the illustrated axons are just schematic, without reflecting the actual number or spatial extent of end-points. **(B)** Examples of axonal arborizations of MOs PT and IT neurons in the striatum ipsilateral to the somata, drawn by using the data downloaded from the MouseLight database (http://ml-neuronbrowser.janelia.org/). Projections onto the horizontal section are drawn. The black lines and red points indicate the axons and axonal end-points, respectively, and the blue cross indicates the center of those end-points (i.e., the point whose coordinates are the means of the *x*, *y*, and *z* coordinates of individual end-points). The horizontal and vertical axes correspond to *x* [medial-lateral (or right-left)] and *z* (anterior-posterior) directions, respectively. The scale bars in the middle indicate 1 mm. The text under each panel describes the following information: ID number in the MouseLight database, with the layer of soma in the cases of IT neurons; Num: number of axonal end-points in the ipsilateral striatum or also in the contralateral striatum in the cases of IT neurons; SD*x*, SD*y*, SD*z*: the standard deviation (SD) of *x*, *y*, and *z* coordinates of the ipsilateral intra-striatal end-points (in [μm]); and SD dist: SD of the distances of ipsilateral intra-striatal end-points from the center of those end-points (in [μm]). **(a)** Examples of PT neurons, whose SD of *x* coordinates of the ipsilateral intra-striatal axonal end-points (corresponding to the medial-lateral (or right-left) direction) are the 2, 4, 6, 8, 10, 12, 14, and 16-th from the smallest one among 16 MOs layer 5 PT neurons (the order is from the top-left to top-right and then bottom-left to bottom-right). **(b)** Examples of IT neurons, whose SDs of *x* coordinates of the ipsilateral intra-striatal axonal end-points are the 10, 22, 34, 46, 58, 70, 82, and 94-th from the smallest one among 94 MOs IT neurons having at least one ipsilateral end-point (the order is from the top-left to top-right and then bottom-left to bottom-right).

We have also examined the spatial extent of the distribution of ipsilateral axonal end-points, measured by the SD of the coordinates or of the distances from the center of end-points. With these measures, we have shown that IT ipsilateral axonal end-points on average have wider spatial distributions than PT end-points, with the difference along the medial-lateral axis most prominent. Distinguishing the subpopulations of MOs IT neurons, we have shown that layer 5 and bilateral-Str-targeting layer 2/3 IT neurons have a wider spatial distribution of ipsilateral axonal end-points than MOs PT neurons, whereas contra-Str-non-targeting layer 2/3 IT neurons are comparable to MOs PT neurons in these measures. Together with the abovementioned results for the number of axonal end-points, and considering that most layer 5 IT neurons target bilateral striatum (36/43 in MOs), it can be said, at least as for MOs, that bilateral-Str-targeting CS neurons generally have more extensive ipsilateral axons than contra-Str-non-targeting CS neurons in terms of the number and the spatial extent of end-points, as summarized in Figure 5A. The larger number and wider spatial extent of axonal end-points of bilateral-Str-targeting than contra-Str-non-targeting CS neurons suggests a possibility that former neurons affect a larger number of striatal neurons than the latter neurons, and functional significance of this would be interesting to explore.

As exemplified in this article, the MouseLight database (Winnubst et al., 2019) is quite useful for testing the issues raised in previous anatomical and morphological studies with a smaller number of neurons. However, an important limitation is that information about synapses is not available in this database, as mentioned before, in contrast to the previous studies that identified individual boutons (Zheng and Wilson, 2002) or even analyzed them by electron microscopy (Kincaid et al., 1998). Instead we analyzed the information about axonal end-points. However, it is not infrequent that a considerable portion of intra-striatal axons do not have any end-point (e.g., AA0182 and AA0011 PT neurons or AA0470 IT neuron in Figure 5B). Some of them could potentially be the traversing parts of axons before arriving at targeting sites where many synapses are made. The findings on spiny or thalamic neurons’ axons in cat visual cortex that the bouton density is twice hither in distal segments than in proximal segments (Anderson et al., 2002) could potentially support our focus on the end-points. Nevertheless, the prevalence of end-point-free axons still casts doubt about whether the number of axonal end-points is well correlated with the number of synapses, and also about whether the spatial extent of axonal end-points well reflects the spatial extent of the entire intra-striatal presynaptic terminals. The latter issue is concerned also by the existence of cases where intra-striatal axons consist of multiple parts that are rather separate (e.g., AA0243 IT neuron in Figure 5B). These issues are expected to be complemented by future studies.

## Data Availability Statement

The datasets analyzed for this study can be found in the MouseLight database (http://ml-neuronbrowser.janelia.org/).

## Author Contributions

YK conceived of analyzing the data of IT and PT neurons in the MouseLight database. All authors designed the analyses and edited the manuscript. KM performed the analyses and drafted the manuscript. SI made the figure of example neurons (Figure 1).

## Conflict of Interest

The authors declare that the research was conducted in the absence of any commercial or financial relationships that could be construed as a potential conflict of interest.
